# Use of multiple indicators multiple causes (MIMIC) method to investigate quantitative inference in socioeconomic determinants on motorcyclist stress

**DOI:** 10.1016/j.mex.2025.103240

**Published:** 2025-02-22

**Authors:** Iqra Mona Meilinda, Sugiarto Sugiarto, Sofyan M. Saleh, Ashfa Achmad

**Affiliations:** a Doctoral Program, School of Engineering, Post Graduate Program, Syiah Kuala University, Banda Aceh, 23111, Indonesia; b Department of Civil Engineering, Universitas Syiah Kuala, Banda Aceh 23111, Indonesia; c Center for Environmental and Natural Resources Research, Universitas Syiah Kuala, Banda Aceh, 23111, Indonesia; d Department of Architecture and Planning, Universitas Syiah Kuala, Banda Aceh, 23111, Indonesia

**Keywords:** Driver stress, MIMIC, Socio-economic, Heart rate variability, Safe driving, Multiple Indicators Multiple Causes (MIMIC)

## Abstract

Research has shown that driving-related stress plays a significant role in causing traffic accidents, either directly or indirectly. Motorcyclists often engage in risky driving behaviors due to elevated stress levels. This study investigates the influence of socioeconomic factors on driving stress among motorcyclists. Data were gathered from 50 participants, with heart rate (HR) recorded using the Polar Vantage V2 device. Heart rate variability (HRV) was analyzed in both time and frequency domains using Kubios HRV software. The study employed the Multiple Indicators Multiple Causes (MIMIC) model to explore the associations between socioeconomic factors and driving stress. The results indicate that variables such as age, gender, education level, occupation, income, driving experience, and travel purpose significantly affect stress levels across both HRV domains. These findings highlight the importance of addressing motorcyclist stress through targeted interventions, including educational programs and policy measures that regulate driving duration. Such strategies are particularly vital in developing countries to reduce stress and improve road safety. This research provides a foundation for developing practical solutions aimed at minimizing driving stress and enhancing the well-being of motorcyclists in high-risk environments.

• A MIMIC model was applied to analyze the relationship between stress variables in the time and frequency domains based on HRV data.
• The model identified significant causal relationships, emphasizing the pivotal role of socioeconomic factors in influencing motorcyclists' driving stress.
• The model demonstrated strong statistical performance with key indicators: chi-square = 38.749, GFI = 0.958, CFI = 0.982, AGFI = 0.893, TLI = 0.961, and RMSEA = 0.057, confirming its robustness and reliability.

## Graphical abstract

**Figure:**
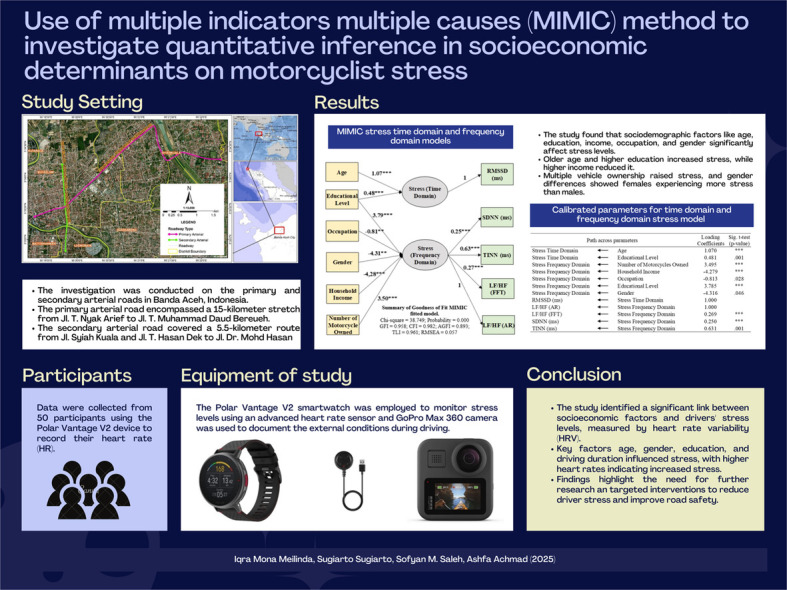

Specifications table

**Table:** 

Subject area:	Engineering
More specific subject area:	Travel Behavior / Driving Stress
Name of your method:	Multiple Indicators Multiple Causes (MIMIC)
Name and reference of original method:	P. P. Thwe, T. Yamamoto, H. Sato, and T. Morikawa, Analysis of driving stress on various roadway conditions in Myanmar by using heart rate variability, Asian Transport Studies, 4 (4) (2017) 663–679
Resource availability:	Information on all resources needed to reproduce this procedure is included in the present article

## Background

Previous research linked driving stress to traffic accidents [[1], [2], [3]]. showing a correlation between physiological responses and stress. Driving stress, associated with heart rate and respiration, was a key traffic safety indicator for motorcyclists [4]. Highest heart rates occurred in commercial zones and road curvatures, with land-use factors like offices and schools affecting stress levels [5]. Previous research has predominantly focused on evaluating driver stress through psychological, physical, and physiological responses [[6], [7]]. Psychological evaluations have been the most frequently utilized methodology for assessing stress, predominantly employing self-report measures or questionnaires administered subsequent to driving sessions [8]. Nevertheless, post-experiment questionnaires may inadequately capture a driver's real-time emotional states, potentially resulting in biased results. Additionally, certain studies have endeavored to assess driver stress using physiological indicators, including facial expressions and vehicle dynamic data [9]. A substantial body of research has used physiological methods, such as cardiac activity, electrodermal activity, and respiratory patterns, to evaluate stress. Heart rate variability (HRV) has emerged as a reliable, noninvasive technique for assessing driver stress, offering insights into autonomic nervous system fluctuations regulating cardiac function [[10], [11], [12], [13]].

Study by [14], regulates involuntary physiological processes such as heart rate to maintain homeostasis and adapt to environmental stimuli [15]. Electrocardiography (ECG) records heart activity [16], enabling the extraction of heart rate variability (HRV) data to analyze cardiac autonomic modulation. Frequency-domain HRV metrics, such as high-frequency (HF) and low-frequency (LF) power, offer insights into parasympathetic and sympathetic activity, respectively. Furthermore, time-domain and nonlinear heart rate variability (HRV) metrics have been employed to characterize various driver states [[16], [17]]. Moreover, heart rate variability (HRV) is influenced by multiple factors, including age, gender, cardiorespiratory fitness, circadian rhythms, and psychological stress [[18], [19], [20], [21]]. Research showed that HRV characteristics, like the LF/HF ratio, changed under stress or fatigue [22]. Furthermore, age significantly affects traffic accidents, with younger drivers showing more antisocial behaviors and slower hazard perception due to inexperience. Machin and Sankey [23] or difficulty regulating emotions [24]. Conversely, elderly drivers showed unsafe behaviors in velocity regulation and lane transitions. Age was considered a socioeconomic factor impacting driving behavior and stress [25].

In a study conducted by [26], younger drivers displayed more hostile and anxious behaviors; males were aggressive and risky, and females were more cautious and nervous. Gender differences also appeared in cognitive tasks like mental rotation and spatial orientation [27]. A limited number of studies have focused on the cognitive demands of driving. Research indicates that males show a greater tendency for high-risk driving behaviors, while females struggle with vehicle control and navigation in complex traffic, contributing to gender-based differences in driving behavior [[27], [28]]. This study examines how socioeconomic factors, such as age, gender, education, income, driving experience, and daily travel habits, influence driving stress among motorcycle riders. By analyzing these variables, the research aims to deepen understanding of the link between socioeconomic characteristics and driving behavior, providing a foundation for strategies to reduce driving stress in urban environments.

## Method details

### Area of Study

**Fig. 1:**
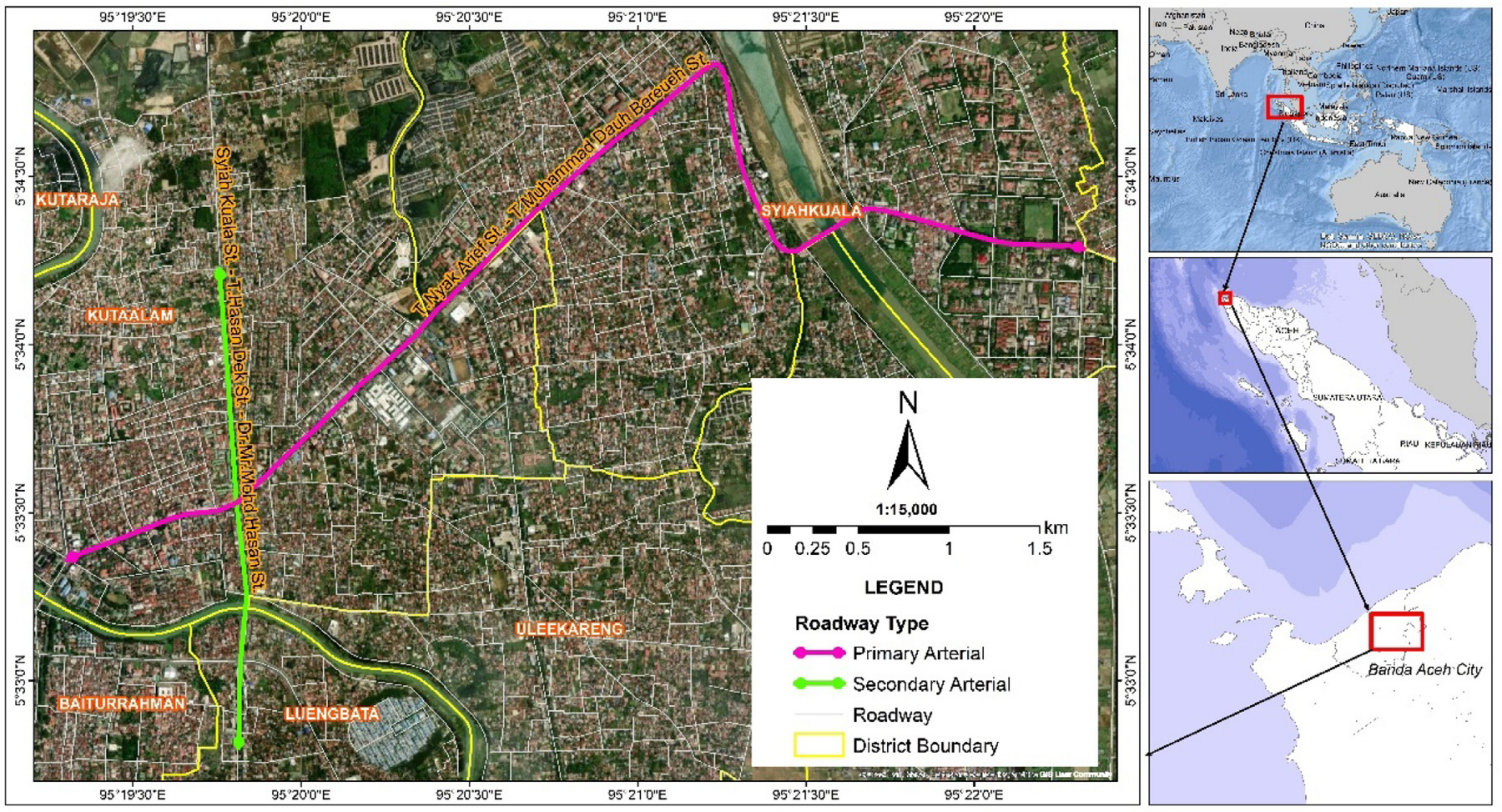
Location of experiments.

The investigation was conducted on the primary and secondary arterial roads in Banda Aceh, Indonesia. The primary arterial road encompassed a 15-kilometer stretch from Jl. T. Nyak Arief to Jl. T. Muhammad Daud Bereueh, while the secondary arterial road covered a 5.5-kilometer route from Jl. Syiah Kuala and Jl. T. Hasan Dek to Jl. Dr. Mohd Hasan in Fig. 1. The sample comprised 50 participants. Data were collected during peak hours (morning, afternoon, and evening) in accordance with participants' availability and consent. Participants were randomly selected to ensure a balanced representation of students and workers, as well as an equal distribution of male and female riders. All participants were in good health and possessed valid motorcycle driving permits.

### Equipment of study

**Fig. 2:**
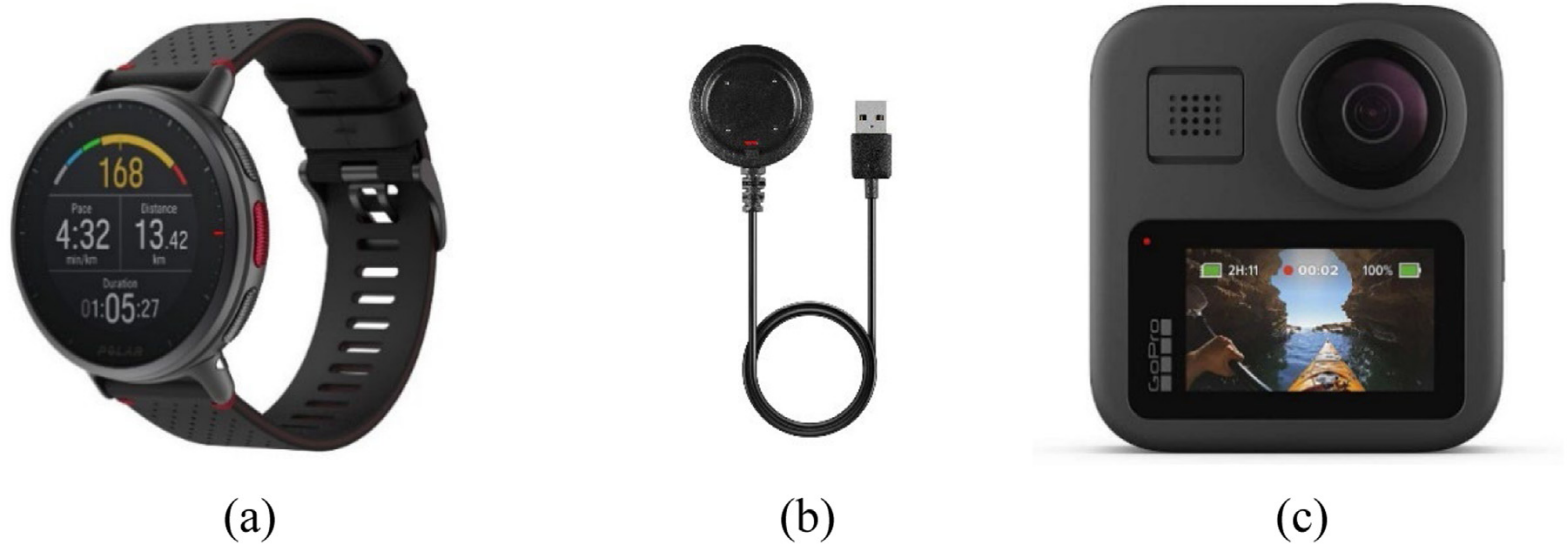
(a) Vantage V2 polar watch; (b) Polar USB cable; (c) GoPRO MAX 360 camera.

The driving experiment utilized two primary instruments: a heart rate sensor integrated into the Polar Vantage V2 Multisport Smartwatch and the GoPro Max 360 camera. The Polar Vantage V2 smartwatch was employed to monitor stress levels using an advanced heart rate sensor. The participants wore smartwatches on their wrists throughout the experiment. Baseline heart rate data were recorded 10 min prior to the driving session to represent the rest condition, and continuous heart rate data were collected while the participants navigated a predefined route. These signals were transmitted to the smartwatch and subsequently extracted for analysis using a Polar Flow application. Concurrently, a GoPro Max 360 camera was used to document the external conditions during driving. The camera captured detailed visuals of road classifications, adjacent land-use types, traffic settings, real-time traffic conditions, and roadway geometry. This dual-instrument approach facilitated a comprehensive analysis of physiological stress responses in relation to environmental factors affecting driving experience (Fig. 2).

### Data collection method

**Fig. 3:**
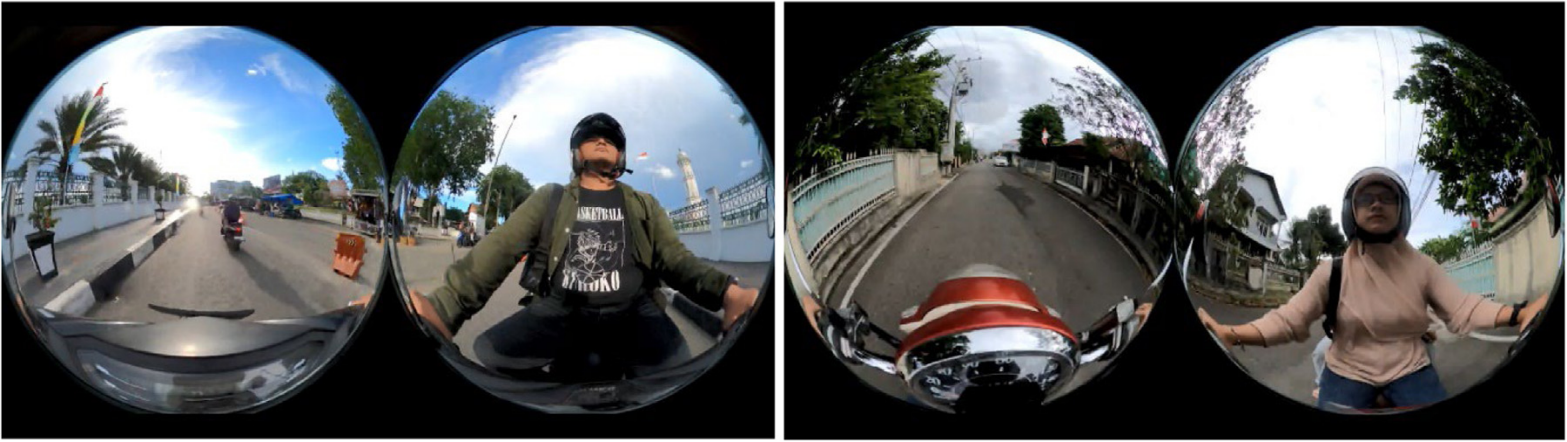
Participants involved as research samples.

The data collection process was initiated by instructing participants to navigate predetermined routes. Two categories of routes were incorporated into the study: a primary arterial road encompassing Jalan T. Nyak Arief to Jalan T. Muhammad Daud Bereueh, and a secondary arterial road comprising Jalan Syiah Kuala and Jalan T. Hasan Dek to Jalan Dr. Mr. Mohd Hasan. Fifty participants were involved in the study, with 25 individuals allocated to each route. Data collection was conducted with three to four participants per day, ensuring a systematic approach to testing and observation (Fig. 3).

Prior to the driving experiment, participants attended a briefing session in which they were informed about the study's objectives, procedures, and safety requirements. These included possessing a valid driving license (SIM C) and utilizing protective helmets. Participants also completed a questionnaire to provide sociodemographic data, including information on age, gender, education level, occupation, travel purpose, average daily trips, household income, driving experience, daily driving duration, number of motorcycles owned, and possession of a driving license. To ensure that the participants were in a relaxed state prior to the experiment, they wore a Polar Vantage V2 smartwatch for 10 min before driving to record baseline heart rate data. During the experiment, the participants operated their vehicles at an average speed of 60 km/h along designated routes. Upon completion of the drive, the heart rate data recorded by the Polar Vantage V2 smartwatch were extracted using the Polar Flow application. These data were subsequently organized and analyzed using the Multiple Indicators Multiple Causes (MIMIC) method.

### MIMIC model framework

In this context, Exploratory Factor Analysis (EFA) investigated the relationship between stress variables in the time and frequency domains and variable X, which comprised the heart rate variability (HRV) parameters. These included the standard deviation of normal-to-normal RR intervals (SDNN), root mean square of successive differences in RR intervals (RMSSD), triangular interpolation of NN intervals (TINN), and measures of autonomic balance, such as the ratio of low-frequency (LF) to high-frequency (HF) power using fast Fourier transform (FFT) and LF/HF ratio using autoregressive (AR) modeling. Conversely, Confirmatory Factor Analysis (CFA) examined the relationship between the stress variables (time and frequency domains) and indicator Y, which encompassed the socioeconomic characteristics of the drivers. These characteristics included age, gender, education level, occupation, travel purpose, average daily trips, household income, driving experience, daily driving time, number of motorcycles owned, and possession of driving license. Throughout the modeling process, all independent and dependent variables were represented as dummy variables to encode categorical data for inclusion in the model. In the multiple linear regression analysis, dummy variables were constructed to represent distinct categories within the independent variables and indicators [[29], [30]].

## Method validation

### Socio-economic data distributions

Table 1 presents the distribution of the 50 participants included in the study, encompassing a range of demographic and driving-related characteristics. The gender distribution was balanced, with 60 % male and 40 % female participants. The age groups were as follows: 22 % were aged 17–19 years, 72 % were aged 20–29 years, and 6 % were aged 30–39 years. Education levels varied, with 38 % holding primary school or equivalent qualifications, 58 % having undergraduate degrees, and 4 % holding postgraduate degrees. Regarding employment status, 2 % were government employees, 12 % were private employees, 2 % were self-employed, 24 % were students, and 60 % fell into the “other” category. Furthermore, travel purposes included business/work (10 %), education (40 %), shopping (26 %), and vacations (16 %). The daily trip frequency ranged from 1 to more than 5 trips, distributed as follows: 2 %, 40 %, 32 %, 14 %, and 12 %, respectively. Monthly household income was categorized into five ranges: 1–2.9 million IDR (4 %), 3–4.9 million IDR (28 %), 5–6.9 million IDR (28 %), 7–9.9 million IDR (36 %), and more than 10 million IDR (4 %).

**Table 1:** Variable percentage of driving stress of urban arterial roads.

Socio-Economic Characteristics of participants		Number of participants
Gender	Male	30
	Female	20
Ages	Ages of 17–19	11
	Ages of 20–29	36
	Ages of 30–39	3
Education Level	Primary school graduates/equivalent	19
	Undergraduate graduates	29
	Postgraduate graduates	2
Types of Occupations	Civil Servants/Military Personnel/Police Officers/State-Owned Enterprises (SOEs) Employees	1
	Private employees	6
	Self-employed	1
	Students	12
	Others	30
Travelling Purposes	Business/work	9
	Education	20
	Shopping	13
	Vacation	8
Frequent Trips	1	1
	2	20
	3	16
	4	7
	>5	6
Monthly Income	<1 million,	0
	1–2.9 million	2
	3–4.9 million	14
	5–6.9 million	14
	7–9.9 million	18
	>10 million	2
Driving Time Per Day	<1 hour	10
	1–3 hours	37
	3–5 hours	2
	5–8 hours	0
	> 8 hours	1
Driving Experience	1–5 years	0
	5–10 years	14
	10–15 years	30
	15–20 years	4
	> 20 years	2
Vehicle Ownership	1 unit	14
	2 units	11
	3 units	20
	4 units	4
	> 4 units	1

Regarding mobility attributes, the daily driving time was categorized into four groups: less than 1 h (20 %), 1–3 h (74 %), 3–5 h (4 %), and more than 8 h (2 %). Driving experience was distributed as follows: 5–10 years (28 %), 10–15 years (60 %), 15–20 years (8 %), and > 20 years (4 %). Vehicle ownership ranged from one to more than four units, with respective distributions of 28 %, 22 %, 40 %, 8 %, and 2 %. All participants possessed a valid two-wheeled driving license (SIM C) to ensure compliance with the licensing requirements.

### Heart rate variability distributions

The parameters analyzed in this study were SDNN, RMSSD, TINN, LF/HF (FFT), and LF/HF (AR). These metrics were selected because of their well-established roles as indicators of autonomic nervous system function and heart rate variability (HRV), both of which reflect the physiological stress experienced by drivers. Data were processed using Kubios HRV Standard 3.5.0, a widely recognized tool for HRV analysis. Tables 2 and 3 present the heart rate (HR) data for drivers on primary and secondary arterial roads, revealing specific mean values. These findings elucidate the physiological responses of drivers to varying road conditions and demonstrate the relationship between the road type and stress level.

**Table 2:** HRV indicators on primary arterial roads.

Participants ID	MEAN HR	MEAN RR	SDNN (ms)	RMSSD (ms)	PNN50 (%)	TINN (ms)	LF/HF (FFT)	LF/HF (AR)
1	94.379	636.683	7.882	8.690	0.000	28.473	5.523	36.114
2	91.215	658.599	10.537	5.877	0.784	44.045	8.432	10.773
3	80.802	743.025	14.410	5.547	0.170	29.591	5.454	8.645
4	84.370	712.963	13.043	6.200	0.291	48.364	9.079	10.164
5	106.752	563.859	13.255	4.519	0.064	35.091	9.595	36.255
6	105.408	569.502	9.767	4.570	0.055	37.318	14.127	15.672
7	95.000	632.855	10.126	6.125	0.607	41.409	9.574	14.421
8	89.832	668.211	8.985	5.663	0.166	32.682	7.203	14.910
9	94.133	641.550	14.120	8.718	0.841	56.091	5.428	30.252
10	92.647	649.240	13.745	7.237	0.334	52.773	8.242	27.020
11	90.645	663.439	9.521	5.363	0.186	34.273	9.708	12.606
12	98.600	608.744	8.914	4.478	0.065	34.136	11.844	53.208
13	95.142	631.484	9.801	5.396	0.394	31.091	8.766	26.546
14	77.444	775.503	11.216	7.040	0.260	43.273	5.933	12.984
15	99.366	604.881	11.802	6.282	0.340	45.727	7.462	13.666
16	100.250	599.171	8.213	4.151	0.113	32.227	9.916	12.856
17	97.523	616.003	8.815	4.440	0.143	32.352	8.013	15.939
18	100.158	599.888	12.796	6.309	0.233	46.682	13.465	28.530
19	99.507	604.887	9.609	4.955	0.284	39.545	7.654	14.008
20	103.781	579.488	8.345	4.318	0.164	31.773	8.481	16.907
21	92.665	649.221	9.239	5.604	0.065	35.227	9.514	16.691
22	87.670	687.242	14.229	7.679	0.528	53.682	7.882	16.241
23	95.605	628.120	8.236	4.514	0.035	32.955	7.323	8.890
24	79.331	799.948	28.582	19.735	0.684	127.227	6.943	34.958
25	83.917	718.071	17.195	11.137	0.848	66.773	5.858	6.260

**Table 3:** Heart Rate Variability (HRV) Indicators on Secondary Arterial Roads.

Participants ID	MEAN HR	MEAN RR	SDNN (ms)	RMSSD (ms)	PNN50 (%)	TINN (ms)	LF/HF (FFT)	LF/HF (AR)
1	85.825	700.461	6.479	3.959	0.000	28.067	3.975	15.671
2	104.718	533.978	6.899	3.684	0.032	24.600	4.664	8.952
3	74.797	804.625	12.877	7.928	0.199	46.800	8.846	11.640
4	80.039	751.414	8.780	5.345	0.392	30.867	3.900	7.847
5	78.542	765.511	9.626	5.050	0.032	31.933	5.161	39.737
6	94.294	640.583	12.139	7.160	0.588	39.933	3.388	14.698
7	109.443	4044.842	5.797	10.187	0.000	19.400	2.109	9.927
8	92.615	649.525	8.455	4.671	0.000	29.800	4.699	22.565
9	83.927	717.305	7.884	5.215	0.000	27.667	2.860	9.031
10	108.453	553.627	7.587	4.097	0.370	25.400	2.500	11.059
11	104.715	574.185	5.830	3.046	0.000	19.800	3.561	12.745
12	79.074	767.541	9.044	5.404	0.000	29.867	12.543	12.032
13	87.846	685.810	15.720	7.175	1.176	36.933	6.057	12.566
14	96.297	625.488	7.425	11.778	0.476	33.733	3.054	13.790
15	93.333	644.479	6.682	4.073	0.100	21.600	3.335	8.022
16	80.137	750.314	10.138	5.910	0.033	34.670	2.043	9.782
17	104.068	578.509	9.045	4.732	4.364	39.800	8.844	8.522
18	85.338	709.805	10.070	6.089	0.475	39.200	8.509	11.635
19	97.309	617.407	7.894	4.420	0.000	25.533	2.810	8.789
20	94.904	638.481	8.929	4.864	0.000	30.933	2.638	11.494
21	104.027	579.713	5.413	3.432	0.000	20.400	1.134	6.220
22	90.020	668.559	10.591	5.147	0.021	37.533	6.988	20.698
23	83.427	722.107	12.348	5.896	0.000	50.200	3.841	37.896
24	96.850	621.254	7.249	10.032	0.000	23.467	3.198	11.748
25	77.001	733.388	9.044	5.404	0.000	29.867	12.543	12.539

The Mean HR (average heart rate) in Table 2 values among drivers on primary arterial roads exhibited variability, ranging from 77.444 bpm to 106.752 bpm. Most drivers demonstrated a Mean HR exceeding 90 bpm, indicating a relatively elevated level of cardiovascular activity, potentially attributable to the physical or cognitive demands associated with driving. Drivers with higher Mean HR values, such as IDs 5, 6, 16, 18, and 20, likely experienced increased levels of physiological arousal or tension compared with those with lower Mean HR values, such as IDs 14 and 24. Although the Mean HR values for all drivers remained within the normal resting heart rate range (60–100 bpm) as defined by [[30], [31], [32]], several drivers exhibited HR values exceeding 100 bpm, potentially indicating elevated stress or anxiety levels. These elevated HR levels may be attributed to driving conditions or environmental factors, thus underscoring the potential influence of external stimuli on the physiological responses during driving.

The Mean HR (average heart rate) in Table 3 values for participants in this study ranged from 74.797 bpm to 109.443 bpm. Most participants exhibited Mean HR values exceeding 90 bpm, indicating relatively high cardiovascular activity, which was likely influenced by driving demands. Drivers with higher Mean HR values, such as IDs 2, 7, 10, 11, 17, and 21, surpassed 100 bpm, potentially indicating elevated stress or anxiety levels during the driving task. These elevated heart rates may be attributed to factors such as traffic conditions, environmental stressors, or individual physiological responses to driving. Conversely, participants with lower Mean HR values, such as IDs 3, 5, 12, and 25, displayed moderate cardiovascular responses. This suggests a less stressful driving experience and reduced exposure to stress-inducing factors.

### MIMIC model result

The MIMIC model was employed to investigate the relationship between stress variables in the time and frequency domains and heart rate variability (HRV) parameters (X variables). These parameters encompassed the standard deviation of normal-to-normal RR intervals (SDNN), the root mean square of successive differences in RR intervals (RMSSD), triangular interpolation of the NN interval histogram (TINN), and the low-frequency (LF)/high-frequency (HF) ratio derived through fast Fourier transform (FFT), representing the balance between sympathetic and parasympathetic nervous system activity. Additionally, the model examined the association of these stress variables with socioeconomic indicators of the drivers (Y variables), including age, gender, education level, occupation, travel purpose, average daily trips, household income, driving experience, daily driving duration, number of motorcycles owned, and possession of a driving license.

**Fig. 4:**
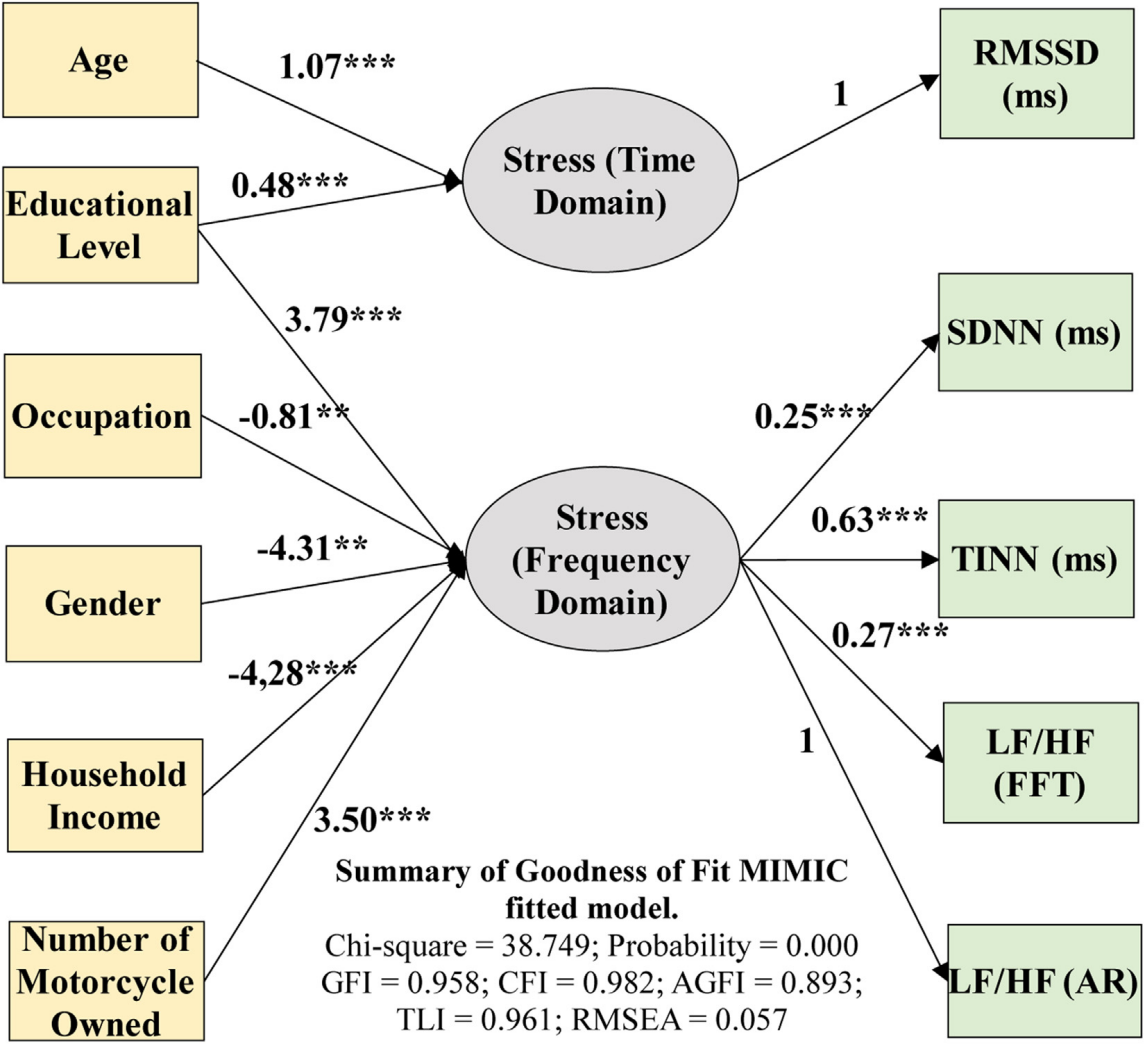
MIMIC stress time domain and frequency domain models.

Among the factors analyzed, significant effects were observed primarily in the drivers' socioeconomic characteristics, including age, gender, education level, occupation, household income, and the number of motorcycles owned. These variables were found to have a substantial impact on the stress levels experienced by drivers in both time and frequency domains. Fig. 4 presents the hypothetical model with generalized values, illustrating the influence of drivers' socioeconomic characteristics on vehicular stress across time and frequency domains.

The model depicts the causal relationships between these variables, with significance levels represented by unidirectional arrows. Statistical significance was denoted as *p < 0.10, **p < 0.05, and ***p < 0.01, highlighting the critical role of socioeconomic factors in understanding driving stress. These findings emphasize that personal and economic factors are key determinants of stress, offering valuable insights into traffic management and the development of driver safety interventions. The chi-square correlation was employed to evaluate the overall model fit, yielding a value of 38.749. The MIMIC stress model demonstrated an acceptable fit in both time and frequency domains, with goodness-of-fit (GOF) statistics as follows: Comparative Fit Index (CFI) = 0.982, Adjusted Goodness-of-Fit Index (AGFI) = 0.893, Tucker Lewis Index (TLI) = 0.961, and Root Mean Square Error of Approximation (RMSEA) = 0.057. These results indicate robust model performance in capturing the relationships between socioeconomic factors and driving stress (Table 4).

**Table 4:** Calibrated parameters for time domain and frequency domain stress model.

Path across parameters			Loading Coefficients	Sig. t-test (p-value)
Stress Time Domain	←	Age	1.070	***
Stress Time Domain	←	Educational Level	0.481	.001
Stress Frequency Domain	←	Number of Motorcycles Owned	3.495	***
Stress Frequency Domain	←	Household Income	-4.279	***
Stress Frequency Domain	←	Occupation	-0.813	.028
Stress Frequency Domain	←	Educational Level	3.785	***
Stress Frequency Domain	←	Gender	-4.316	.046
RMSSD (ms)	←	Stress Time Domain	1.000	
LF/HF (AR)	←	Stress Frequency Domain	1.000	
LF/HF (FFT)	←	Stress Frequency Domain	0.269	***
SDNN (ms)	←	Stress Frequency Domain	0.250	***
TINN (ms)	←	Stress Frequency Domain	0.631	.001

## Discussion

The influence of sociodemographic parameters on stress in both time and frequency domains revealed several significant relationships. In the time-domain stress analysis, age had a substantial impact on stress levels (standardized coefficient = 1.070, *p* < 0.01, highly significant). This finding suggests that time-domain stress increases with advancing age. These results are consistent with previous studies, such as those by [[33], [34]], which reported a similar trend of stress levels increasing with age. Factors contributing to this pattern may include a decline in physical health, alterations in cognitive processes and responses to stress, and an increasing burden on daily responsibilities. Older individuals may experience elevated stress levels in traffic situations because of reduced physical resilience and heightened concerns regarding road safety. Additionally, education level significantly affected time-domain stress (standardized coefficient = 0.481, *p* < 0.01). While individuals with higher education levels tend to manage their time more efficiently, they remain susceptible to stress, particularly in scenarios that require complex decision making or the fulfillment of intricate responsibilities.

In the frequency domain, the number of motorcycles exhibited a highly significant positive correlation with stress levels (standardized coefficient = 3.495, *p* < 0.01). This association may be attributed to the increased responsibilities concomitant with multiple vehicle ownership. Additional motorcycles often necessitate higher maintenance expenditures, increased time allocation for upkeep, and logistical complexities, all of which contribute to elevated stress levels. Conversely, household income demonstrated a highly significant negative correlation with frequency-domain stress (standardized coefficient = −4.279, *p* < 0.01), these findings align with previous research by [35], indicating that elevated income levels correlate with decreased stress. This reduction in stress is likely attributable to the enhanced financial capacity of higher-income individuals to address stressors such as vehicle maintenance expenses or the challenges posed by suboptimal road conditions. Furthermore, increased income provides greater flexibility in managing unforeseen circumstances, thereby mitigating stress. These findings align with previous research by [36], which demonstrated a negative correlation between higher income levels and the risk of depression. This study indicates that increased financial resources contribute to the reduction of economic stressors and enhance access to resources that support mental well-being, thereby mitigating stress and other psychological burdens.

Occupations demonstrated a small but statistically significant negative impact on the frequency domain stress (standardized coefficient = −0.813, *p* < 0.05). This finding suggests that occupation may contribute to stress reduction, potentially because of the structure and routine it provides. However, the relatively minor effect size indicates that other factors, such as workload or work environment, may exert a more substantial influence on stress levels [37]. Conversely, educational level demonstrated a highly significant positive impact on frequency-domain stress (standardized coefficient = 3.785, *p* < 0.01). This finding suggests that individuals with higher educational attainment may experience increased cognitive demands or greater occupational responsibilities, which can increase their stress levels. Higher education frequently correlates with more complex tasks, increased responsibilities, and elevated expectations in both the professional and personal spheres, potentially contributing to heightened stress levels.

Gender also demonstrated a significant negative effect on frequency domain stress (standardized coefficient = −4.316, *p* < 0.05). In a sample comprising 60 % male and 40 % female respondents, the results indicated that male participants experienced lower levels of frequency domain stress compared to their female counterparts. This finding suggests that females may exhibit heightened sensitivity to stressors, such as road conditions or traffic situations, which is potentially attributable to variations in psychological resilience or societal expectations regarding safety. These findings align with prior research by [[25], [37]], which underscored the significant role of gender in influencing stress responses in comparable contexts.

## Conclusions

This investigation identified a statistically significant correlation between drivers' socioeconomic factors and the stress levels experienced under varying road conditions. Stress was quantified using heart rate variability (HRV) indicators, including SDNN, RMSSD, TINN, and the LF/HF ratio, which reflect the balance between sympathetic and parasympathetic nervous system activities. The analysis revealed that variables such as age, gender, education level, occupation, trip purpose, and driving duration significantly influenced drivers' stress levels in both time and frequency domains. Variations in the average heart rate (HR) among drivers were correlated with stress levels, with elevated HR values generally indicating increased stress. The findings from the MIMIC model further corroborate the critical role of socioeconomic factors in influencing drivers' stress levels.

Future research could build upon these findings by conducting longitudinal studies to examine how drivers' stress levels evolve over time in response to changes in socioeconomic conditions, driving habits, and exposure to diverse road environments. Furthermore, external environmental factors, such as air quality, noise levels, and weather conditions, can complement heart rate variability (HRV)-based analyses. Expanding the study population to include a more diverse range of drivers, such as commercial, older, and novice drivers, would provide more comprehensive insights into the variations in stress responses across different demographic groups. Assessing the efficacy of targeted interventions, such as stress management training, driver assistance technologies, or road design modifications, could potentially contribute to the reduction of stress levels and the enhancement of safety. These findings emphasize the significance of incorporating socioeconomic considerations into traffic management policies and driver safety initiatives to formulate more effective strategies aimed at mitigating driver stress, improving safety, and optimizing road conditions.

## Limitations

Not applicable.

## Ethics statements

Attached. All participants provided written informed consent prior to participating in the study.

## CRediT author statement

**Iqra Mona Meilinda**: Conceptualization, software, data curation, writing—original draft preparation, writing—review and editing, formal analysis. **Sugiarto Sugiarto**: Methodology, investigation, funding acquisition. **Sofyan M. Saleh**: Validation, funding acquisition, resources, supervision. **Ashfa Achmad**: Visualization, validation, project administration.

## Declaration of competing interest

The authors declare that they have no known competing financial interests or personal relationships that could have appeared to influence the work reported in this paper.

## Data Availability

Data will be made available on request.
